# NIRS-KIT: a MATLAB toolbox for both resting-state and task fNIRS data analysis

**DOI:** 10.1117/1.NPh.8.1.010802

**Published:** 2021-01-25

**Authors:** Xin Hou, Zong Zhang, Chen Zhao, Lian Duan, Yilong Gong, Zheng Li, Chaozhe Zhu

**Affiliations:** a Beijing Normal University, IDG/McGovern Institute for Brain Research, State Key Laboratory of Cognitive Neuroscience and Learning, Beijing, China; b Beijing Normal University at Zhuhai, Center for Cognition and Neuroergonomics, State Key Laboratory of Cognitive Neuroscience and Learning, Zhuhai, China; c Beijing Normal University, Center for Collaboration and Innovation in Brain and Learning Sciences, Beijing, China

**Keywords:** near-infrared spectroscopy-KIT, functional near-infrared spectroscopy, resting-state, task activation, data analysis, toolbox

## Abstract

**Significance:** Functional near-infrared spectroscopy (fNIRS) has been widely used to probe human brain function during task state and resting state. However, the existing analysis toolboxes mainly focus on task activation analysis, few software packages can assist resting-state fNIRS studies.

**Aim:** We aimed to provide a versatile and easy-to-use toolbox to perform analysis for both resting state and task fNIRS.

**Approach:** We developed a MATLAB toolbox called NIRS-KIT that works for both resting-state analysis and task activation detection.

**Results:** NIRS-KIT implements common and necessary processing steps for performing fNIRS data analysis, including data preparation, quality control, preprocessing, individual-level analysis, group-level statistics with several popular statistical models, and multiple comparison correction methods, and finally results visualization. For resting-state fNIRS analysis, functional connectivity analysis, graph theory-based network analysis, and amplitude of low-frequency fluctuations analysis are provided. Additionally, NIRS-KIT also supports activation analysis for task fNIRS.

**Conclusions:** NIRS-KIT offers an open source tool for researchers to analyze resting-state and/or task fNIRS data in one suite. It contains several key features: (1) good compatibility, supporting multiple fNIRS recording systems, data formats of NIRS-SPM and Homer2, and the shared near-infrared spectroscopy format data format recommended by the fNIRS society; (2) flexibility, supporting customized preprocessing scripts; (3) ease-to-use, allowing processing fNIRS signals in batch manner with user-friendly graphical user interfaces; and (4) feature-packed data viewing and result visualization. We anticipate that this NIRS-KIT will facilitate the development of the fNIRS field.

## 1 Introduction

As a promising non-invasive neuroimaging technique, functional near-infrared spectroscopy (fNIRS) has seen increasing use in neuroscientific studies over the last decades.^1^^–^^3^ Compared to other neuroimaging techniques, fNIRS has several specific advantages: acceptable spatial resolution; lower cost, silent, higher temporal resolution, lower sensitivity to head motion, and fewer physical constraints compared to functional magnetic resonance imaging (fMRI); portability/mobility and few restrictions on use location. These advantages make it an ideal alternative for detecting human brain activity in both healthy people (from newborns to the elderly) and patients with neurological or psychiatric conditions, in both laboratory and natural contexts. Validation studies using simultaneous fNIRS and fMRI scanning have shown fNIRS to be effective for both task-driven activation detection^4^^–^^8^ and resting-state brain function research.^9^^,^^10^ FNIRS has been successfully applied to various neuroscience domains, including areas such as motor-related studies with large bodily movements, social cognitive neuroscience, and neurodevelopment.^11^^–^^15^ In addition to task-evoked activation studies, fNIRS has also seen increasing use in detecting spontaneous brain activity absent of external stimuli during resting state,^16^^–^^20^ which is particularly useful for several special populations who cannot perform complicated tasks, such as infants and patients with certain deficits.

There are several fNIRS data analysis toolboxes currently available, such as NIRS-SPM,^21^ Homer,^22^ nirsLAB,^23^ FieldTrip,^24^ and POTATo.^25^ (For more software, see the official website of the Society for fNIRS in Ref. 26.) These useful software packages mainly focus on task-driven cortical activation. However, few software packages can assist for resting-state fNIRS studies. FC-NIRS^27^ was recently developed for resting-state fNIRS, supporting functional connectivity (FC) analysis and graph theory-based network analysis. However, it only supports few data sources, which limited its application for resting-state studies severely.

As a fundamental feature of the resting brain, low-frequency fluctuations are closely related to spontaneous neural activity and vary among different brain regions and individuals. Amplitude of low-frequency fluctuation (ALFF)^28^ and fractional ALFF (fALFF)^29^ quantify the amplitude of these low-frequency oscillations and reflect individual differences or dysfunction.^30^^–^^33^ These indices have been supported by several fMRI analysis toolboxes, such as REST,^34^ DPARSF,^35^ and RESTplus,^36^ but have not been supported by any existing fNIRS related toolboxes.

Researchers usually collect data from both task and resting-state modalities in their fNIRS studies to explore the same or related scientific questions.^19^^,^^37^ For example, researchers have used significantly activated regions in task fNIRS analysis to define seed regions for resting-state FC analysis^38^ or used resting-state FC strength to predict task activation.^39^ In this situation, researchers must switch between at least two packages to perform task and resting-state fNIRS analyses. Additionally, different processing methods and parameters between packages make it more difficult to integrate, compare, and explain analysis results in a single study. Therefore, there is an urgent need to develop a comprehensive fNIRS toolbox to perform analysis for both modalities.

We developed and present here a MATLAB toolbox called NIRS-KIT that allows researchers to analyze resting-state and/or task fNIRS data in one suite. With a friendly graphical user interface (GUI), this open source package allows researchers to perform common and necessary fNIRS data analysis steps in an easy way. It has good compatibility and flexibility, supporting multiple fNIRS recording systems and customized preprocessing scripts. It can execute most processing steps in a batch processing model, which processes data from multiple participants simultaneously rather than one-by-one. NIRS-KIT can be freely downloaded from the NIH NeuroImaging Tools & Resources Collaboratory website in Ref. 40. In this tutorial paper, we first introduce the main functionality of NIRS-KIT and then provide step-by-step instructions for users.

## 2 Toolbox Development

**Fig. 1:**
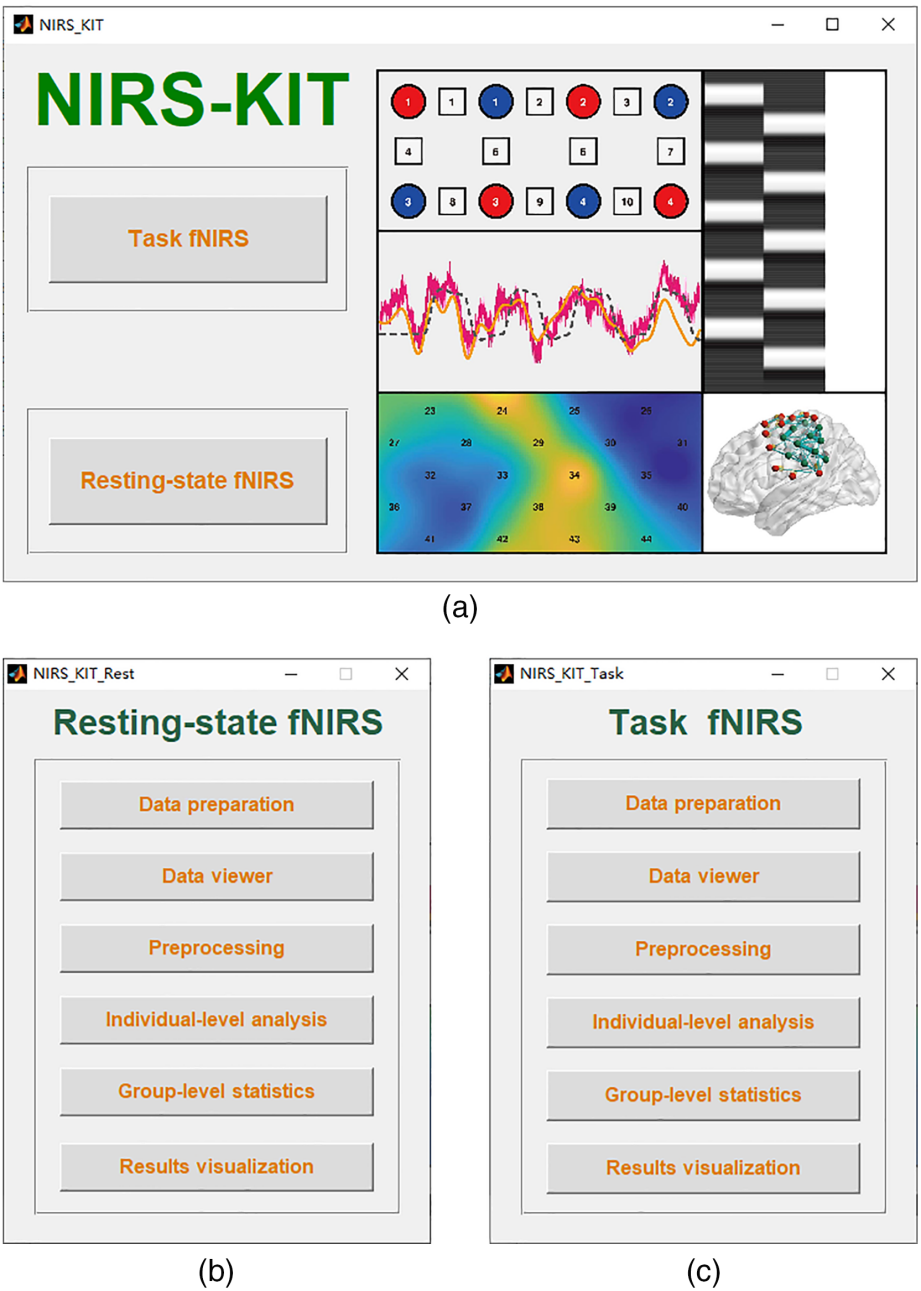
User interfaces of NIRS-KIT: (a) the main interface window of NIRS-KIT; (b) subwindow for the resting-state fNIRS analysis module; and (c) subwindow for the task fNIRS analysis module.

NIRS-KIT [Fig. 1(a)] was developed using MATLAB 2012a (The MathWorks Inc., Natick, MA, USA) as the programming language under a 64-bit Windows 10 (Microsoft Corp., Redmond, WA, USA) environment. NIRS-KIT has been successfully tested under a variety of operating systems with MATLAB installed, including Windows, Linux, and Mac OS.

## 3 General Overview of NIRS-KIT

**Fig. 2:**
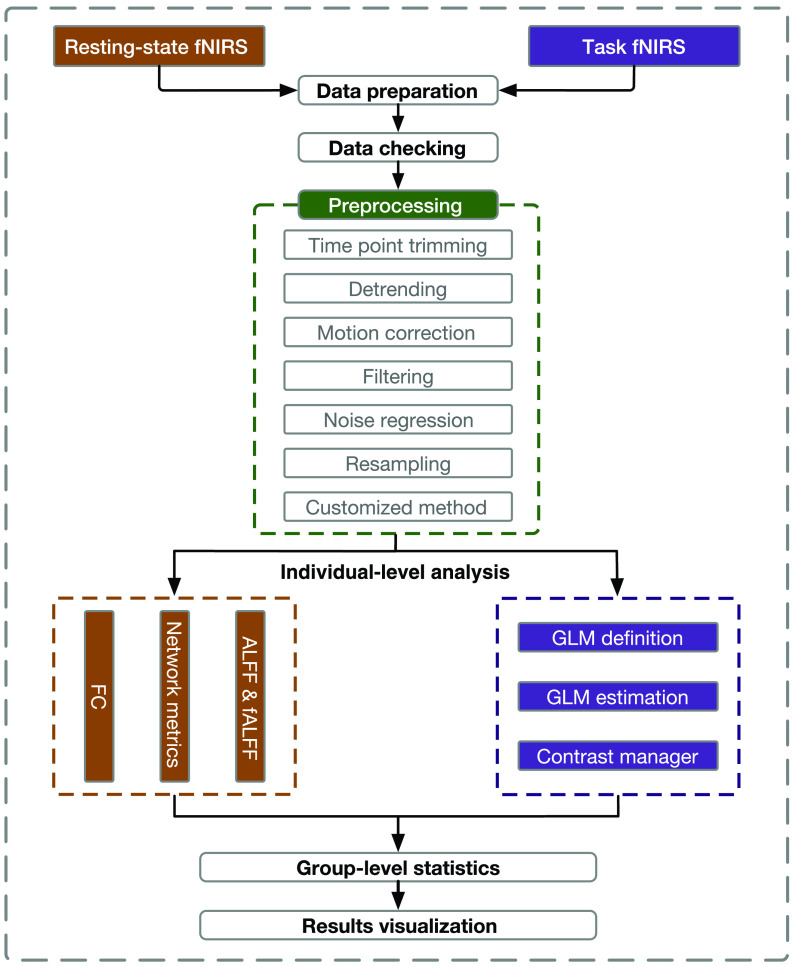
Main processing pipeline in NIRS-KIT. FC, functional connectivity; GLM, general linear model; ALFF, amplitude of low-frequency fluctuation; and fALFF, fractional amplitude of low-frequency fluctuation.

NIRS-KIT has two main analysis modules: resting-state fNIRS module and task fNIRS module [Figs. 1(b) and 1(c)]. The fNIRS data analysis pipeline implemented in NIRS-KIT is illustrated in Fig. 2. This pipeline consists of common and necessary processing steps for fNIRS data analysis, including data preparation, quality control, preprocessing, individual-level analysis, group-level statistics, and results visualization.

For resting-state fNIRS individual-level analysis, FC analysis, ALFF and fALFF, and graph theory-based network analysis to investigate complex topological properties of brain networks (such as local or global efficiency) are supported. In individual-level analysis for task fNIRS, general linear model (GLM) is used to detect task activation.

The following sections will explain these functions and steps in detail, first for the resting-state module and then for specific parts of the task fNIRS module.

## 4 Resting-State fNIRS Analysis

### 4.1 Data Preparation

A variety of data are obtained in an fNIRS experiment, and data first need to be imported into the analysis toolkit. The most important data, the raw fNIRS signal time series, are the relative concentration changes of oxyhemoglobin (HbO), deoxyhemoglobin (HbR), or total hemoglobin (HbT). In addition to signal data series, the fNIRS recording’s spatial information, consisting mainly of probe and channel locations in two-dimensions (2D) or three-dimensions (3D), are also very important for some analyses, results visualization, data sharing, and publication. Thus we designed a NIRS-KIT data format (stored in MATLAB.mat files), which includes not only the time series signals, but also spatial information about probe set geometry and standard coordinates of source, detector, and channel positions in Montreal Neurological Institute (MNI) coordinates.

#### 4.1.1 Preparation of temporal signals (hemoglobin concentrations)

FNIRS optical time course data are obtained from diverse commercial devices in different types or formats. Some devices only output the raw optical intensity data, whereas others support the calculation of the relative hemoglobin concentration change with in-house conversion functions. Different fNIRS recording systems use different output formats (such as .txt and .csv). These inconsistent data sources pose difficulty for following-up analysis, thus a unified format for hemoglobin concentration is need.

**Fig. 3:**
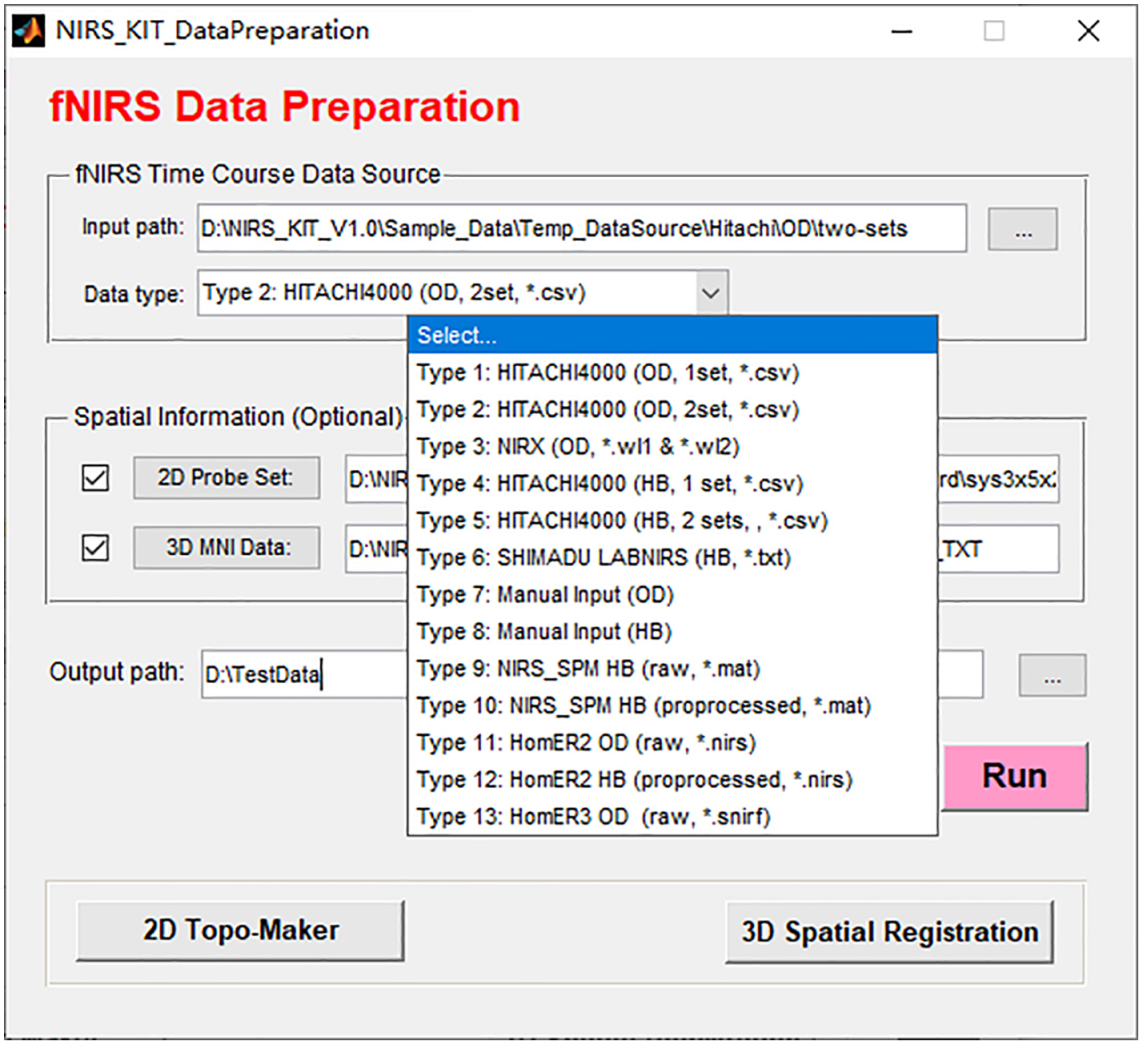
User interface for data preparation module with supported data sources.

NIRS-KIT provides time course preparation functionality for diverse fNIRS data sources (Fig. 3). If the raw optical intensity data are obtained, such as from Hitachi ETG4000/7000 (.csv), or NIRX (.wl1 and .wl2), they can first be converted into optical density data and then converted to concentration changes of HbO and HbR via the modified Beer–Lambert law.^41^ The converted hemoglobin concentration data will be saved in the NIRS-KIT data format. If the converted hemoglobin concentration change data are directly obtained, such as from Hitachi ETG4000/7000 (.csv) or Shimadzu LABNIRS (.txt), they will be reformed and saved in the NIRS-KIT supported format.

If the recording system is not one of the above, NIRS-KIT also provides a manual input function to generate the required data format. Here users just need to reorganize their raw data (optical density data or hemoglobin concentration data) into a specific format (.csv) according to the format in the sample file included in the toolbox.

NIRS-KIT has a good compatibility with two widely used fNIRS data analysis packages. Both raw and preprocessed fNIRS time course data from NIRS-SPM (.mat) or HomER2 (.nirs) can be read and saved in the NIRS-KIT supported data format.

Recently, the Shared Near-Infrared Spectroscopy Format (SNIRF), a new fNIRS data format standard, has been proposed by the fNIRS community recently (see Ref. 26), and adopted by Homer3 and FieldTrip. NIRS-KIT also supports SNIRF as input.

#### 4.1.2 Preparation of topospatial information (probe setup)

The probe setup, consisting of the configuration of the sources, detectors, and measurement channels, provides spatial information that needs to be included in the data file. The topographical geometry of the channels is very useful for checking time course signals for problems and result visualization, especially when adopting a complex or irregular probe design. NIRS-KIT provides several standard probe settings in the software package’s sample folder (including standard 3×3, 3×5, 3×5×2, 3×11, and 4×4 probes). If an appropriate probe setup file does not already exist, users can use the topomaker module (see Fig. 3) to generate, in a simple and flexible way, a customized probe setup file with arbitrary arrangements of sources and detectors.

#### 4.1.3 Preparation for spatial information in standard brain space

Three-dimensional information in standard brain space, consisting of the spatial locations of optodes and channels, is important for result interpretation, visualization in brain space, and publication so as to enable replication and meta-analysis. NIRS-KIT allows spatial registration of NIRS optodes or channels to standard MNI space with the NFRI toolbox developed by Singh et al. (2005).^42^ Two text files for each participant are necessary. One contains fiducial (landmark) coordinates in real space (usually obtained using a 3D digitizer), in which at least four reference positions of the international 10–20 system^43^ are given. The reference positions should be spatially separated, and the combination of five standard positions—Iz (inion), Nz (nasion), AL (left preauricular point), AR (right preauricular point), and Cz (central zero)—are commonly used with good effect. The other text file contains the optode and channel coordinates in real space. After loading the above files, a text file containing MNI coordinates of each channel and optode in standard brain space will be outputted. In addition, NIRS-KIT also reports the structural labels of the brain regions closest to the channel locations in three brain atlases (Brodmann, AAL, and LPBA40 atlases).

### 4.2 Data Previewing and Quality Control

**Fig. 4:**
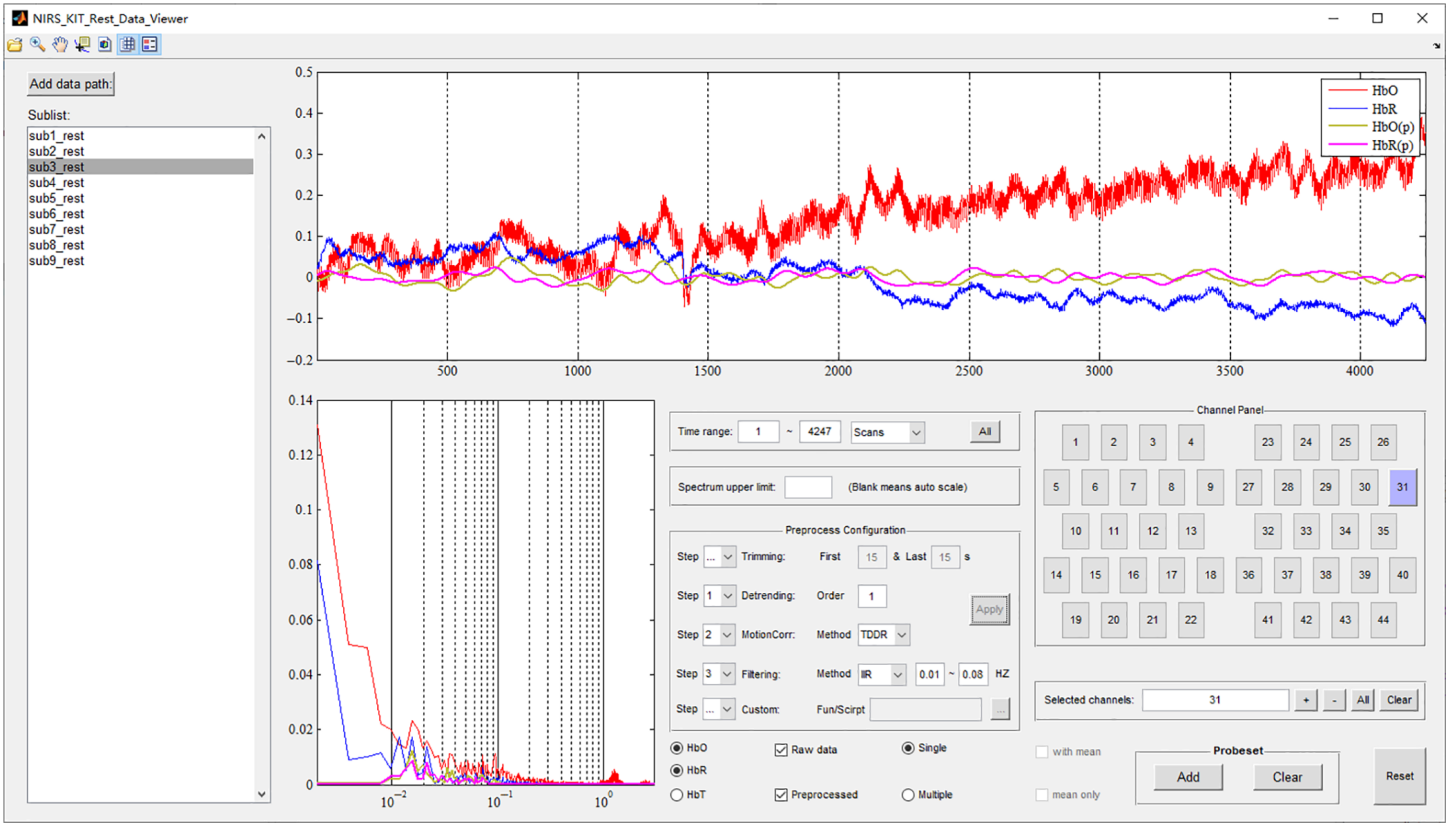
The Data Viewer module for resting-state fNIRS. A 7-min resting-state fNIRS dataset (n=9) using two standard 3×5 probes (total 44 channels) is shown as an example.

After data preparation, the raw fNIRS data should be checked in the time-domain and frequency domain before formal data analysis to examine their characteristics and quality, which help inform choices regarding which participants’ data to exclude due to noise and how parameters should be set in subsequent preprocessing. The Data Viewer (Fig. 4) module of NIRS-KIT provides a variety of visualization functions for plotting individual raw and processed signals, in time or frequency domain.

The Data Viewer module offers abundant and versatile features which include: (1) selection of channels by typing or clicking on the channel in the probe setup; (2) display of both time series and periodogram for selected channels; (3) choosing specific or multiple hemoglobin signal types (HbO, HbR, or HbT); (4) display of signals from multiple channels simultaneously; (5) simultaneously display of raw signals and signals after processing by the preprocessing module (see the following section); and (6) various other features to assist in data examination, such as zoom, pan, data cursor, and change color scheme adjustment.

### 4.3 Preprocessing

**Fig. 5:**
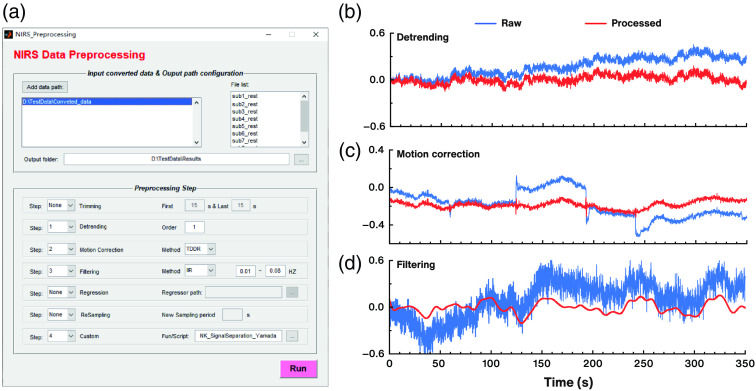
The data preprocessing interface and demonstrations of processing methods on real data. (a) GUI of the data preprocessing module; (b) demonstration of the effect of detrending (linear detrending); (c) demonstration of motion correction using TDDR method; and (d) demonstration of filtering by a third-order IIR Butterworth bandpass filter (0.01 to 0.08 Hz).

The fNIRS data typically undergo a series of preprocessing steps prior to statistical analysis, to minimize the influence of noise and artifacts from exogenous and endogenous sources. The NIRS-KIT preprocessing module [Fig. 5(a)] provides several common preprocessing functions for fNIRS signals, including time point trimming, detrending, motion correction, filtering, noise regression, resampling, and customized processing methods. Researchers can arbitrarily designate the parameters and the order of these steps.

#### 4.3.1 Time point trimming

The first few time points of recordings sometimes need to be discarded due to participants’ process of adaptation to the experimental environment. If this option is selected, NIRS-KIT will delete the specified initial and/or last time periods from the data of each participant.

#### 4.3.2 Detrending

FNIRS signals may suffer from systematic increases or decreases over time due to long-term physiological shifts or instrumental instability. NIRS-KIT uses a polynomial regression model to estimate a linear or non-linear trend and subtracts it from the raw hemoglobin concentration signal [as illustrated in Fig. 5(b)]. The default order of the polynomial model is first order to remove linear detrends from the raw time course.

#### 4.3.3 Motion correction

Two parameter-free motion correction methods are available in NIRS-KIT. One is correlation-based signal improvement (CBSI),^44^ which mainly relies on the assumption that the neural components of HbO and HbR signals are strongly negatively correlated, whereas motion artifacts are positively correlated. The other new method is temporal derivative distribution repair (TDDR),^45^ which is based on robust regression and can effectively remove both spike artifacts and baseline shifts [Fig. 5(c)].

#### 4.3.4 Filtering

The signal of interest usually occupies a frequency range different from interference, such as machinery and physiological noise (e.g., respiration, heart rate, Mayer wave, and very low-frequency oscillations). NIRS-KIT provides three filtering models (high-pass, low-pass, and bandpass filtering) to remove irrelevant low-frequency and/or high-frequency components [as illustrated in Fig. 5(d)]. Three commonly used digital filter types are available: infinite impulse response (IIR) filter, finite impulse response (FIR) filter, and fast Fourier transform-based ideal filter (FFT-based filter). The IIR filter used in NIRS-KIT is a Butterworth filter (default third order). The FIR filter is a Hamming window filter (default 34th order). For the FFT-based filter, time series of each channel are transformed into frequency domain, the frequency-domain signals are filtered and then transformed back to time domain. The default is bandpass filtering (0.01 to 0.08 Hz) using a third-order IIR filter. The filtering model, high- and/or low-frequency thresholds and filter type can be specified by the user according to study objectives and noise characteristics. For resting-state, low-frequency fluctuations (0.01 to 0.08 Hz) have been suggested to be of physiological importance and may reflect spontaneous neural activity.^46^^,^^47^ Note, when performing fALFF analysis, the bandpass filtering should be applied to reserve the entire frequency band of the neural signals,^48^ and frequency range selection can refer to fALFF analysis in fMRI studies (usually 0 to 0.25 Hz).

#### 4.3.5 Noise regression

Undesirable systemic artifacts (especially scalp blood flow) usually contaminate fNIRS functional signals, and how to minimize these sources of interference are still a challenge for fNIRS signal processing. A prominent approach is to use short-distance reference channels (that are sensitive only to signals in the superficial layers of the tissue outside the brain) to record superficial noise,^49^^–^^53^ then use regression to remove that noise from neural recordings. When short-distance channels are used, NIRS-KIT provides a noise regression functionality, in which the signals of short-distance channels can be used as the regressor and be removed. In some cases, however, short-distance channels are not available. In these cases, another promising signal separation method proposed by Yamada et al. (2012)^54^ can be used to separate functional and systemic signals based on their hemodynamic differences. This method has been incorporated into the preprocessing module to remove the unwanted systemic noise (also see Sec. 4.3.7).

#### 4.3.6 Resampling

For large scale, multi-site, or hyper-scanning studies, it is sometimes necessary to resample the fNIRS time series data recorded with different recording systems (with different sampling rates) into one sampling rate prior to subsequent processing. Also raw signals sometimes need to be downsampled for computational and memory efficiency. For these reasons, users can select the resample option, set the desired sampling rate, and NIRS-KIT will resample the time series data.

#### 4.3.7 Customized processing methods

In fNIRS signal preprocessing, users might want to use their own processing methods which are adapted to specific needs. Also fNIRS signal processing methodology is continuously being improved by researchers around the world so that novel and modified processing methods are periodically published. If these methods are not included or have not been updated in NIRS-KIT, users can write customized processing methods as a MATLAB functions, and easily incorporate them into NIRS-KIT via the customized interface [see Fig. 5(a)]. Users can refer to the sample customized processing method file in the package [NK_SignalSperation_Yamada.m, a systemized noise remove method developed by Yamada et al. (2012)^54^ was implemented as an example] to make their own customized function or script.

### 4.4 Individual-Level Analysis

**Fig. 6:**
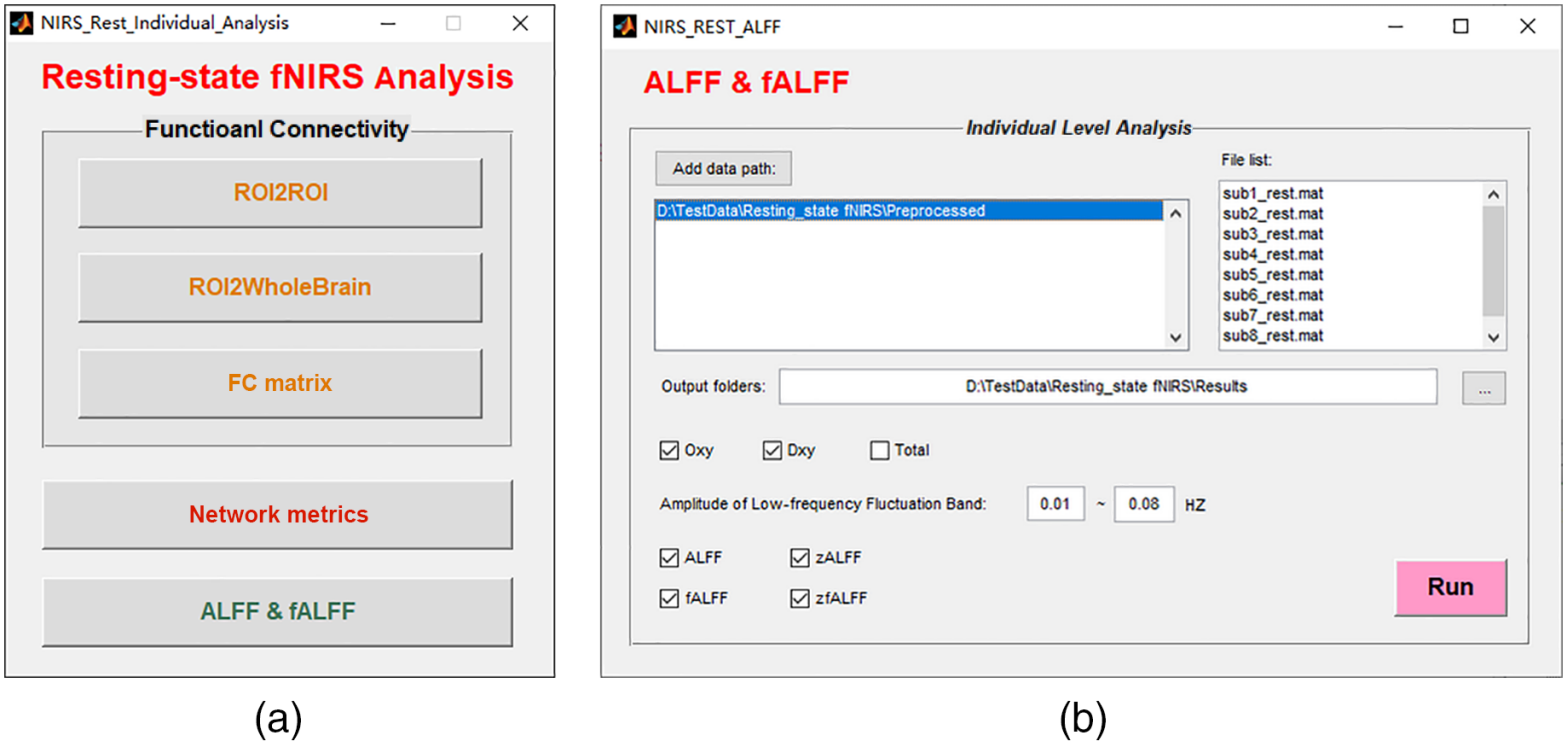
Individual-level analysis interfaces for resting-state fNIRS data in NIRS-KIT: (a) GUI for individual-level analysis of resting-state fNIRS and (b) interface for analyzing ALFF and fALFF.

After preprocessing, individual-level (or first-level) analysis can be performed. Individual-level FC, ALFF, and fALFF can be calculated here for resting-state studies. In addition, NIRS-KIT also calculates FC matrices as input for subsequent graph theory-based network metrics analysis [Fig. 6(a)].

#### 4.4.1 Functional connectivity

Resting-state FC measures temporal correlations in spontaneous fluctuations among spatially distributed brain regions^55^^,^^56^ and has been increasingly used for fNIRS.^17^^,^^57^^,^^58^ NIRS-KIT supports fast ROI2ROI, ROI2Whole-brain, and whole brain (channel-wise) FC analyses.

#### 4.4.2 Network metrics

Network metrics capture complex topological properties of brain networks, such as local and global efficiency^59^^,^^60^ and modularity.^61^ NIRS-KIT allows definition of the network of interest and then calculates the FC matrix of the desired network and saves the result in the file format of Gretna,^62^ a widely used MATLAB-based toolbox for calculating graph-theoretic metrics. Prior to network topological analysis, a thresholding procedure is typically applied to exclude the confounding effects of spurious relationships in interregional connectivity matrices. Two commonly applied thresholding techniques are provided in Gretna: the absolute connectivity strength threshold and relative sparsity threshold (or proportional threshold).^9^^,^^62^^–^^64^ For the former, users need to set a threshold value and the connections with weight greater than the given threshold are retained. For the latter, the threshold value is defined as the ratio of the number of actual edges divided by the maximum possible number of edges in a network. Note that the absolute threshold method may lead to different numbers of network edges across different datasets, which may confound between-group comparisons.^65^

#### 4.4.3 ALFF and fALFF

**Fig. 7:**
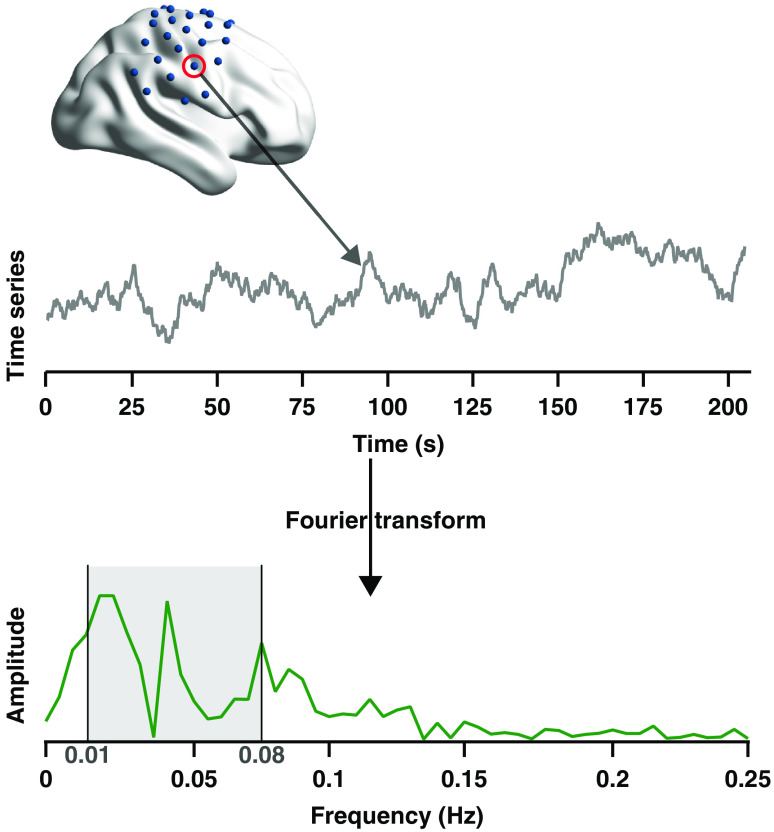
The diagram for computing individual ALFF and fALFF.

ALFF and fALFF, two common resting-state indices characterizing regional spontaneous brain activity,^28^^,^^29^ were not available in any existing fNIRS analysis software. We implemented this functionality in NIRS-KIT [Fig. 6(b)]. The time series signal can be approximated by a finite sum of weighted cosine and sine functions whose frequencies ($f_{k}$) depend on the sampling frequency ($f_{s}$) and the number of collected time points (N):

$$x(t)=a_{0}+\overset{N}{\underset{k=1}{\sum }}[a_{k}\;\cos(2\pi f_{k}t)+b_{k}\;\sin(2\pi f_{k}t)]=a_{0}+\overset{N}{\underset{k=1}{\sum }}A_{k}\;\cos(2\pi f_{k}t-\varphi_{k}),$$ (1)

where $a_{0}$ is the direct-current component [i.e., the mean of the time series x(t)]; $a_{k}$, $b_{k}$ are the weights of the cosine and sine functions; $f_{k}=k\Delta f=k(f_{s}/N)$. The amplitude and phase of k’th frequency component can be expressed as $A_{k}=\sqrt{a_{k}^{2}+b_{k}^{2}}$, $\varphi_{k}=\arctan\;\frac{b_{k}}{a_{k}}$. The time series for each channel is first transformed to a frequency domain to generate the amplitude spectrum by Fourier transform (Fig. 7). Then the averaged amplitude across a specific low-frequency range (usually 0.01 to 0.08 Hz) is calculated as the ALFF of the channel [see Fig. 7 and Eq. (2)]:

$$\mathrm{ALFF}=\frac{1}{N_{k}}\underset{k:f_{k}\in [0.01,0.08]}{\sum }A_{k},$$ (2)

where $N_{k}$ is the number of frequency components within the specific frequency range. The fALFF is a ratio of the total amplitude within the specific low-frequency range (0.01 to 0.08 Hz) to the total fluctuation amplitude within the entire frequency range [see Fig. 7 and Eq. (3)]:

$$\mathrm{fALFF}=\underset{k:f_{k}\in [0.01,0.08]}{\sum }A_{k}/\overset{N}{\underset{k=1}{\sum }}A_{k}.$$ (3)

For each individual, the standardized ALFF and fALFF (zALFF and zfALFF) of a channel (i) are generated by subtracting the global mean value across all channels and then dividing by the standard deviation across all channels [see Eqs. (4) and (5)] to improve the normality of data distribution across participants,^29^^,^^31^ which is more compatible to normal-based group-level statistics:

$${\mathrm{zALFF}}_{i}=\frac{{\mathrm{ALFF}}_{i}-\overset{¯}{\mathrm{ALFF}}}{\sigma \mathrm{ALFF}},$$ (4)

$${\mathrm{zfALFF}}_{i}=\frac{{\mathrm{fALFF}}_{i}-\overset{¯}{\mathrm{fALFF}}}{\sigma \mathrm{fALFF}}.$$ (5)

### 4.5 Group-Level Statistics

**Fig. 8:**
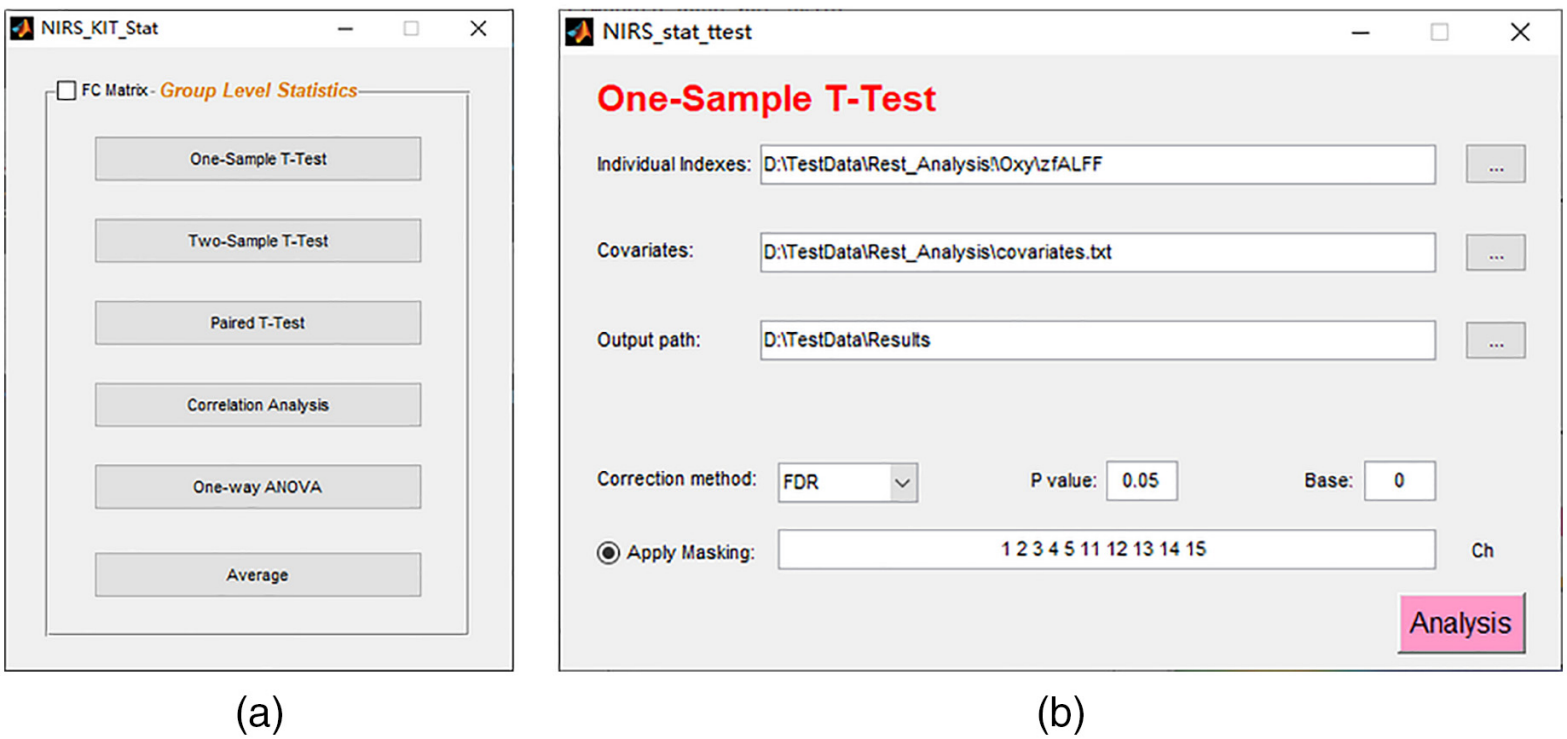
Group-level analysis interfaces: (a) the main GUI for group-level statistics and (b) one-sample t-test is shown as an example.

Group-level analyses consist of comparing differences or computing correlations across multiple participants and making population inferences. Several popular statistical parametric models are available in NIRS-KIT for group-level analysis [see Fig. 8(a)], including one-sample t-test, two-sample t-test, paired t-test, correlation analysis, ANOVA (independent or repeated measurement), and average of individual indices. Covariates other than condition (e.g., age, gender, or training time) can be added into these statistical models [Fig. 8(b)].

To assess statistical significance in the context of multiple tests, the false discovery rate^66^^,^^67^ and Bonferroni correction to control familywise error rate are supported [Fig. 8(b)]. Multiple comparison correction can be set to be performed within certain channels of interest by applying a specified mask [Fig. 8(b)].

Even though several statistical models are provided in NIRS-KIT, several external statistics packages are recommended for more complex statistical analysis, such as the Statistical Package for the Social Sciences (SPSS Inc., Chicago, IL, USA), Statistical Analysis System (SAS Institute Inc., Cary, NC, USA), and R.^68^

### 4.6 Results Visualization

Finally, NIRS-KIT provides plentiful visualization functions in both 2D and 3D to visualize individual-level and group-level results. Because of different ways to organize results and different display requirements, the visualization module is introduced in terms of two approaches, channel-wise visualization, and connectivity matrix visualization.

#### 4.6.1 Channel-wise visualization

**Fig. 9:**
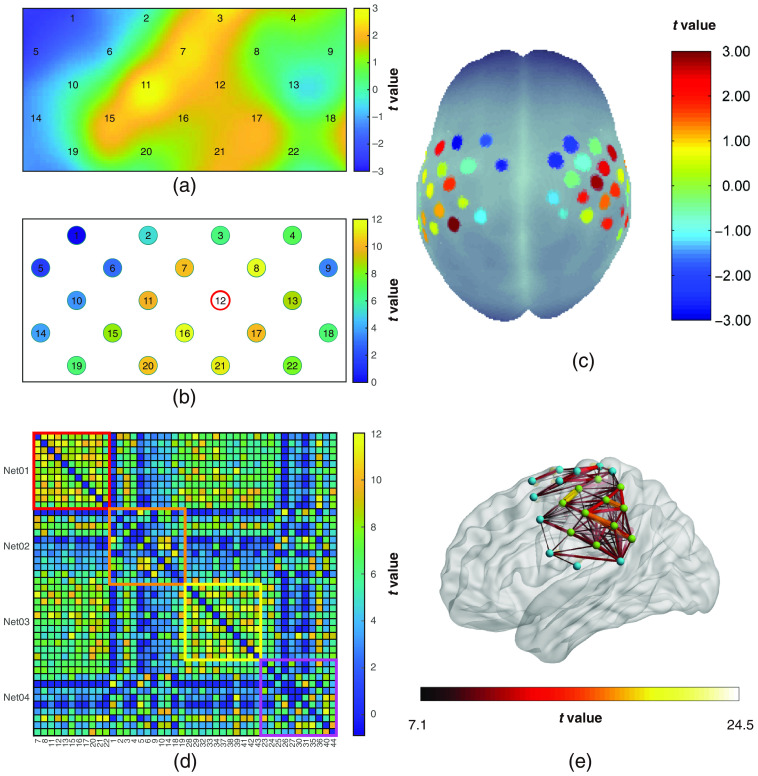
Examples of visualization for resting-state analysis results: (a) group-level zfALFF map (interpolation mode); (b) ROI2Whole FC map (non-interpolation mode, and channel 12 is the seed channel); (c) non-interpolated channel-wise 3D visualization for group-level zfALFF result using the NFRI toolbox; (d) 2D whole brain FC matrix map; and (e) 3D visualization of whole brain connectivity matrix by BrainNet Viewer using the node and edge files generated by NIRS-KIT. Note: All above color bars represent group-level (n=9) one-sample t-test statistical values, and the color axis limits are set for illustration only. In A and B panels, only the left hemisphere probe (channels 1 to 22) is displayed to reduce clutter.

In ALFF/fALFF and ROI2WholeBrain FC, each channel has a single scalar representing the group-level statistical value or individual-level index. Hence we use channel-wise visualization to display these results. For channel-wise 2D visualization, the probe setup (see Sec. 4.1) is required. A blank canvas with channel locations is generated according to the provided probe geometry. Then color values are mapped with interpolation [Fig. 9(a)] or without [Fig. 9(b)]. For ROI2Whole-brain FC, non-interpolated topographical visualization is recommended, and the toolbox will automatically identify the ROI channels and mark them with red circles [Fig. 9(b)]. For channel-wise 3D visualization, the MNI coordinates of each channel are needed for 3D visualization in standard brain space. In NIRS-KIT, three non-interpolated and one interpolated channel-wise 3D visualizations are provided. The first non-interpolated 3D visualization is mapping the channel-wise statistical values on to the standard brain space using the nfri_mni_plot function in the NFRI toolbox.^42^^,^^69^^,^^70^ The second is to project the analysis result of each measurement channel directly on to a standard surface template (e.g., ICBM152), set the display parameters (such as, the sphere size and colorbar limits), and then output the resulting figure. The last, more flexible approach, is to output a brain image in the widely used NIFTI format. Then users can load and plot this image with several other brain visualization toolboxes, such as MRIcroGL^71^ and Surfice.^72^ For interpolated channel-wise 3D visualization, the EasyTopo toolbox provided by Tian et al. (2013)^73^ is used. Here the stereotaxic coordinates of the brain surface and fNIRS channels are converted into spherical coordinates, where 2D angular interpolation of the channel-wise data is implemented to obtain a topographic image of brain activation in the latitude–longitude space. Then the interpolated image is projected back onto the original brain surface. The first non-interpolated 3D visualization using the NFRI toolbox was illustrated in Fig. 9(c). The other three visualization types will be shown in Sec. 5.6 for displaying the task fNIRS analysis results.

#### 4.6.2 Connectivity matrix visualization

The result of whole brain FC is saved as an n×n matrix (n is the number of channels), where each value in the matrix represents the FC between a paired channels or group-level statistic’s value of each FC. For 2D visualization of whole brain FC matrix, users can choose to only display the significant edges by setting a threshold p-value, define the displayed subnetworks, and arbitrarily reorder the channels [Fig. 9(d)]. For 3D visualization of whole brain FC matrix, an Excel (Microsoft Corp., Redmond, WA, USA) file containing MNI coordinates of each channel (see sample files in the package) is need to be loaded, then NIRS-KIT will output a node file and an edge file that can be loaded and visualized by the most existing brain connectome visualization toolkits [Fig. 9(e)], such as BrainNet Viewer.^74^

Several visualization options are available, such as setting display modes, color bar limits, and p-value thresholds.

## 5 Task fNIRS Analysis Module

### 5.1 Data Preparation

There is no difference between task fNIRS data preparation and resting-state fNIRS data preparation (see Sec. 4.1).

### 5.2 Data Previewing and Quality Control

**Fig. 10:**
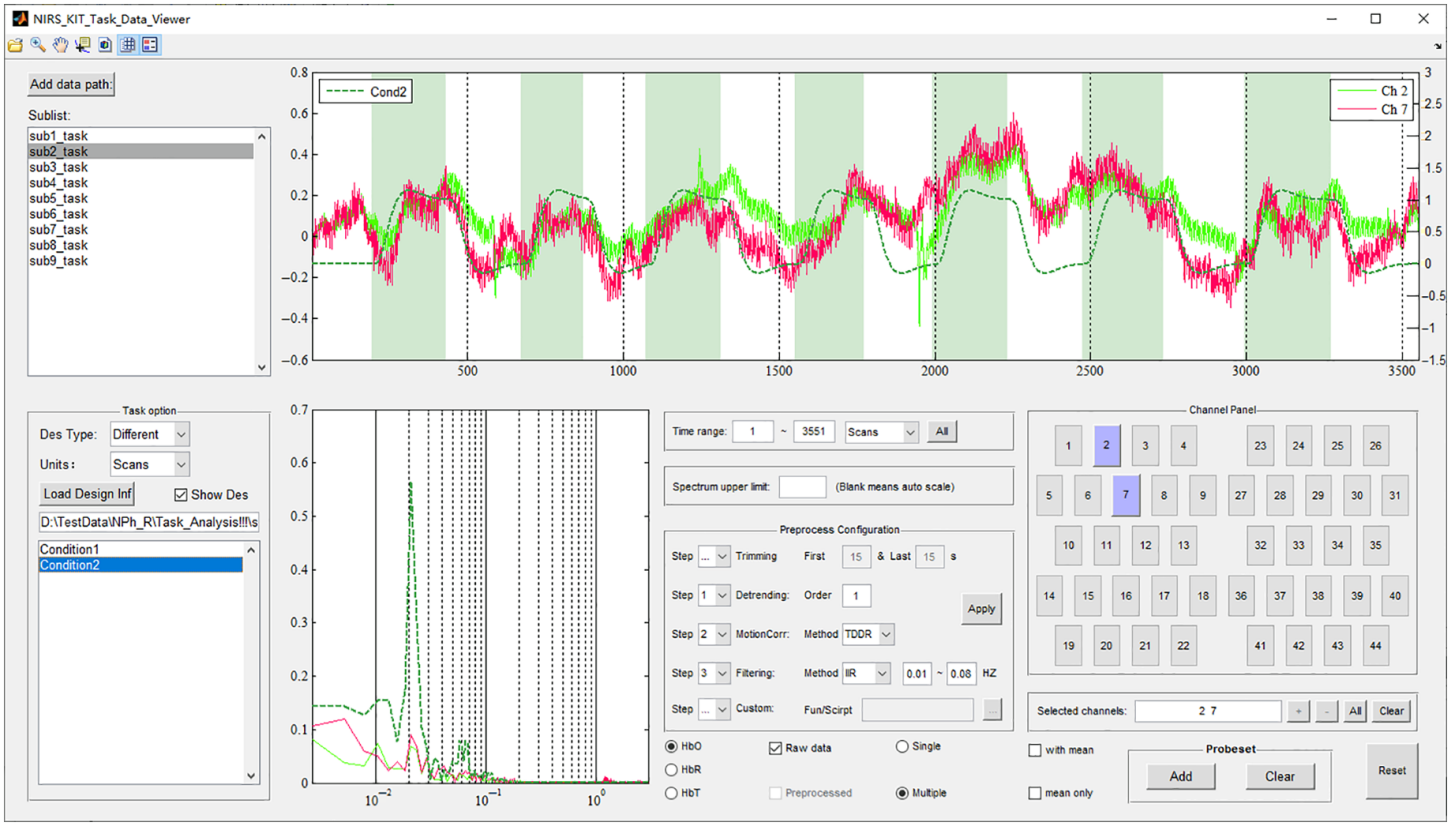
The Data Viewer module for task fNIRS. Task design information is shown in the task option panel (lower left). Dashed green lines indicate reference signal time series (upper right) or the periodogram (lower middle) of the selected task condition.

A specific panel is provided in the Data Viewer module for task fNIRS (Fig. 10). This panel allows stacked display of time series and periodograms of reference signals from the experiment’s protocol. The task-related information (stimuli onset and duration) can be defined by manual input or loading a corresponding task design information file. Users can select single- or multiple-experimental conditions/task types to be displayed.

### 5.3 Preprocessing

Task fNIRS data preprocessing is similar to that of resting-state fNIRS (see Sec. 4.3). For task-based data preprocessing, the frequency components related to the task protocol should not be removed, regardless of which filter is used.

### 5.4 Individual-Level Analyses

After preprocessing for task fNIRS data, individual-level analysis to detect channel-wise task-evoked neural activation can be done via the widely used mass univariate statistical method based on GLMs.^75^ The routine individual-level statistical analysis of task fNIRS data comprises of the following steps: (1) specification of a GLM, which considers the observed hemodynamic signal (dependent variable) as a linear combination of regressors of interest (task variables), nuisance covariates (such as the superficial noise measured by short-distance channels), and an error term. For GLM specification, the canonical hemodynamic response function implemented in SPM is used to construct the reference time series representation from task variables; (2) estimation of GLM parameters on a channel-by-channel basis, which finds the activation beta value (weight coefficient in the linear model) for each experimental condition; and (3) calculation of the condition-wise effects of interest using user-defined contrast vectors as the input for subsequent group-level inference.

**Fig. 11:**
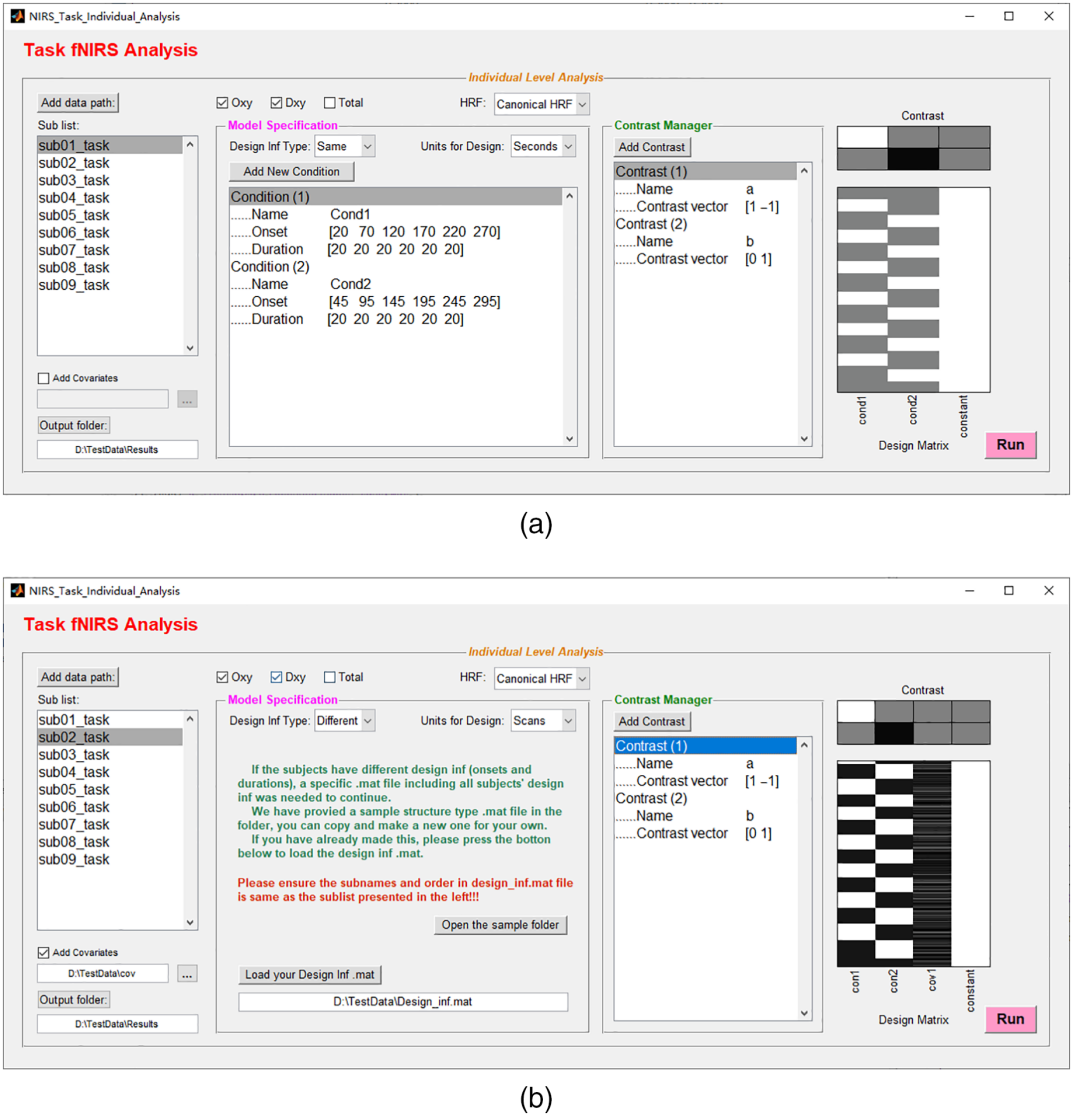
Individual-level analysis interfaces for task fNIRS. (a) The individual-level analysis interface for task fNIRS with manual input of task design (when design is the same for all participants). (b) The individual-level analysis interface for task fNIRS with different designs per participants; one column of randomly generated covariates is added into the GLM as an illustration.

NIRS-KIT supports all the above steps in an easy-to-use, batch processing manner in the Task Individual Analysis module (Fig. 11). There are two ways to input the experimental design information for model specification. If the onset time and duration of each event/block are the same across all participants, the task design information can be entered manually [Fig. 11(a)]. If the design information varies across participants, the design matrix can be constructed by loading a prepared .mat file containing the required experimental design information (names of experimental conditions, onset times of each condition, and event or epoch durations) for each participant [Fig. 11(b)].

### 5.5 Group-Level Statistics

There is no difference with resting-state fNIRS group-level statistical analysis (see Sec. 4.5).

### 5.6 Results Visualization

**Fig. 12:**
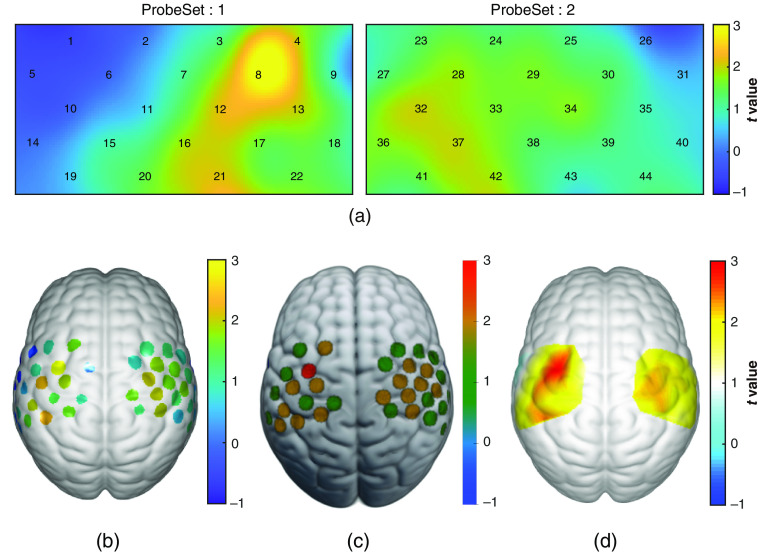
Example visualizations for task fNIRS analysis results: (a) 2D group-level statistical map for task activation (interpolation mode); (b) non-interpolation 3D visualization on a standard brain surface; (c) visualization using MRIcroGL via overlaying the NIFTI file generated by NIRS-KIT; and (d) interpolated 3D visualization on a standard brain surface using EasyTopo. All colors represent the t-statistic values of group-level one-sample t-test.

For task fNIRS activation results, each channel has a single-value representing the group-level statistic’s value, thus channel-wise result visualization should be used for visualization (see Sec. 4.6). Here group-level statistical results from a finger tapping task fNIRS study (n=9) with two standard 3×5 probes are shown for illustration. One 2D [Fig. 12(a)] and three 3D [Figs. 12(b)–12(d)] visualization types are shown.

## 6 Discussion

We developed a comprehensive and versatile MATLAB toolbox called NIRS-KIT, suitable for both resting-state and task fNIRS data analysis. It covers common and necessary fNIRS data analysis steps, including data preparation, quality control, preprocessing, individual-level and group-level analyses, as well as results visualization, in a GUI-based, easy-to-use, batch-processing-supported software suite. We believe NIRS-KIT provides researchers an alternative toolbox for performing complex fNIRS data analysis and will facilitate the advancement of human brain function research.

NIRS-KIT is an integrated platform that supports analysis for both resting-state and task fNIRS data. In recent years, in addition to task-evoked activation studies, fNIRS has also been increasingly used to detect the spontaneous brain activity pattern in resting state without external stimuli. However, few packages support comprehensive analysis for resting-state fNIRS. NIRS-KIT provides related researchers as powerful analysis to perform resting-state fNIRS data analysis. On the other hand, sometimes researchers will collect both task and resting-state fNIRS data in a single study. Prior to NIRS-KIT, the existing fNIRS analysis toolboxes handled one type of experiment or the other. Using NIRS-KIT, researchers can complete fNIRS data analysis of both modalities in one software suite.

NIRS-KIT has good compatibility with multiple data sources and flexibility in usage. First, it supports a variety of the raw data obtained directly from several widely used fNIRS devices, and the shared data format (SNIRF) proposed by the fNIRS community. In addition, it also supports the raw or processed data output from two widely used analysis software (NIRS-SPM and Homer2) as the input. If the data format is not yet support, users can manually reformat the data to a relativity simple text format supported by NIRS-KIT in an easily way. Furthermore, this platform also allows add-ons to incorporate customized processing methods into the preprocessing procedure. Additionally, it supports batch data analysis, which decreases repetitive operations, time cost, and the probability of mistakes, especially when dealing with a large sample with complex processing protocol.

It has been noted that there are still no standard processing procedures in the field of fNIRS, and high heterogeneity of signal processing methods and parameter settings may lead to biased results and undermine the reproducibility of and comparability between studies.^76^^,^^77^ In NIRS-KIT, several mainstream and validated preprocessing methods^53^^,^^78^ are provided, which has the potential to reduce the impacts of heterogeneity to a certain extent. However, more efforts should be taken by researchers around the world to build standardized processing procedures, and processing methods and parameters should also be reported in more detail in publications.

Since NIRS-KIT development is at an early state, improvements are still required. For example, an alternative to FC, effective connectivity mainly depicts the causal influence that one neural system exerts over another.^79^ Effective connectivity has been applied in several fNIRS studies.^80^^–^^82^ In addition, although multiple popular group-level statistical models are provided, individual-level statistical tests are not supported in the current version of NIRS-KIT. We plan to incorporate these functionalities to future versions of NIRS-KIT. Furthermore, some of our research group’s on-going work will be added to NIRS-KIT, e.g., blind source separation (BSS) for separating unrelated physiological noise (such as scalp blood flow) from neural related signals.^37^^,^^83^^,^^84^ Six BSS algorithms (PCA,^85^ SOBI,^86^ JADE,^87^ Fast-ICA,^88^ Infomax-ICA,^89^ and ERBM-ICA^90^) are planned for inclusion.

## References

[1] Ferrari M. Quaresima V., “A brief review on the history of human functional near-infrared spectroscopy (fNIRS) development and fields of application,” Neuroimage 63(2), 921–935 (2012). NEIMEF 1053-8119 10.1016/j.neuroimage.2012.03.049 22510258

[r2] Scholkmann F. et al., “A review on continuous wave functional near-infrared spectroscopy and imaging instrumentation and methodology,” Neuroimage 85, 6–27 (2014). NEIMEF 1053-8119 10.1016/j.neuroimage.2013.05.004 23684868

[3] Strangman G. Boas D. A. Sutton J. P., “Non-invasive neuroimaging using near-infrared light,” Biol. Psychiatry 52(7), 679–693 (2002). BIPCBF 0006-3223 10.1016/S0006-3223(02)01550-0 12372658

[4] Cui X. et al., “A quantitative comparison of NIRS and fMRI across multiple cognitive tasks,” Neuroimage 54(4), 2808–2821 (2011). NEIMEF 1053-8119 10.1016/j.neuroimage.2010.10.069 21047559 PMC3021967

[r5] Sato H. et al., “A NIRS-fMRI investigation of prefrontal cortex activity during a working memory task,” Neuroimage 83, 158–173 (2013). NEIMEF 1053-8119 10.1016/j.neuroimage.2013.06.043 23792984

[r6] Wijeakumar S. et al., “Validating an image-based fNIRS approach with fMRI and a working memory task,” Neuroimage 147, 204–218 (2017). NEIMEF 1053-8119 10.1016/j.neuroimage.2016.12.007 27939793

[r7] Huppert T. J. et al., “A temporal comparison of BOLD, ASL, and NIRS hemodynamic responses to motor stimuli in adult humans,” Neuroimage 29(2), 368–382 (2006). NEIMEF 1053-8119 10.1016/j.neuroimage.2005.08.065 16303317 PMC2692693

[8] Strangman G. et al., “A quantitative comparison of simultaneous BOLD fMRI and NIRS recordings during functional brain activation,” Neuroimage 17(2), 719–731 (2002). NEIMEF 1053-8119 10.1006/nimg.2002.1227 12377147

[9] Duan L. Zhang Y.-J. Zhu C.-Z., “Quantitative comparison of resting-state functional connectivity derived from fNIRS and fMRI: a simultaneous recording study,” Neuroimage 60(4), 2008–2018 (2012). NEIMEF 1053-8119 10.1016/j.neuroimage.2012.02.014 22366082

[10] Sasai S. et al., “A NIRS–fMRI study of resting state network,” Neuroimage 63(1), 179–193 (2012). NEIMEF 1053-8119 10.1016/j.neuroimage.2012.06.011 22713670

[11] Pinti P. et al., “The present and future use of functional near-infrared spectroscopy (fNIRS) for cognitive neuroscience,” Ann. N. Y. Acad. Sci. 1464(1), 5–29 (2020). ANYAA9 0077-8923 10.1111/nyas.13948 30085354 PMC6367070

[r12] Vanderwert R. E. Nelson C. A., “The use of near-infrared spectroscopy in the study of typical and atypical development,” Neuroimage 85, 264–271 (2014). NEIMEF 1053-8119 10.1016/j.neuroimage.2013.10.009 24128733 PMC3910372

[r13] Quaresima V. Ferrari M., “Functional near-infrared spectroscopy (fNIRS) for assessing cerebral cortex function during human behavior in natural/social situations: a concise review,” Organ. Res. Methods 22(1), 46–68 (2019).1094-4281 10.1177/1094428116658959

[r14] Yücel M. A. et al., “Functional near infrared spectroscopy: enabling routine functional brain imaging,” Curr. Opin. Biomed. Eng. 4, 78–86 (2017). 10.1016/j.cobme.2017.09.011 29457144 PMC5810962

[15] Quaresima V. Ferrari M., “A mini-review on functional near-infrared spectroscopy (fNIRS): where do we stand, and where should we go?” Photonics 6(3), 87 (2019). 10.3390/photonics6030087

[16] Niu H. He Y., “Resting-state functional brain connectivity: lessons from functional near-infrared spectroscopy,” Neuroscientist 20(2), 173–188 (2014). 10.1177/1073858413502707 24022325

[r17] Zhu H. et al., “Decreased functional connectivity and disrupted neural network in the prefrontal cortex of affective disorders: a resting-state fNIRS study,” J. Affect. Disord. 221, 132–144 (2017). JADID7 10.1016/j.jad.2017.06.024 28645025

[r18] Nguyen T. et al., “Exploring brain functional connectivity in rest and sleep states: a fNIRS study,” Sci. Rep. 8(1), 16144 (2018). SRCEC3 2045-2322 10.1038/s41598-018-33439-2 30385843 PMC6212555

[r19] Lu C.-M. et al., “Use of fNIRS to assess resting state functional connectivity,” J. Neurosci. Methods 186(2), 242–249 (2010). JNMEDT 0165-0270 10.1016/j.jneumeth.2009.11.010 19931310

[20] Hu Z. et al., “Applications of resting-state fNIRS in the developing brain: a review from the connectome perspective,” Front. Neurosci. 14, 476 (2020).1662-453X 10.3389/fnins.2020.00476 32581671 PMC7284109

[21] Ye J. C. et al., “NIRS-SPM: statistical parametric mapping for near-infrared spectroscopy,” Neuroimage 44(2), 428–447 (2009). NEIMEF 1053-8119 10.1016/j.neuroimage.2008.08.036 18848897

[22] Huppert T. J. et al., “HomER: a review of time-series analysis methods for near-infrared spectroscopy of the brain,” Appl. Opt. 48(10), D280–98 (2009). APOPAI 0003-6935 10.1364/AO.48.00D280 19340120 PMC2761652

[23] Xu Y. Graber H. L. Barbour R. L., “nirsLAB: a computing environment for fNIRS neuroimaging data analysis,” in Biomed. Opt., BM3A.1 (2014).

[24] Oostenveld R. et al., “FieldTrip: open source software for advanced analysis of MEG, EEG, and invasive electrophysiological data,” Comput. Intell. Neurosci. 2011, 1–9 (2011). 10.1155/2011/156869 21253357 PMC3021840

[25] Sutoko S. et al., “Tutorial on platform for optical topography analysis tools,” Neurophotonics 3(1), 010801 (2016). 10.1117/1.NPh.3.1.010801 26788547 PMC4707558

[26] The Society for functional Near Infrared Spectroscopy, https://fnirs.org.

[27] Xu J. et al., “FC-NIRS: a functional connectivity analysis tool for near-infrared spectroscopy data,” Biomed Res. Int. 2015, 248724 (2015). 10.1155/2015/248724 26539473 PMC4619753

[28] Yu-Feng Z. et al., “Altered baseline brain activity in children with ADHD revealed by resting-state functional MRI,” Brain Dev. 29(2), 83–91 (2007). 10.1016/j.braindev.2006.07.002 16919409

[29] Zou Q.-H. et al., “An improved approach to detection of amplitude of low-frequency fluctuation (ALFF) for resting-state fMRI: fractional ALFF,” J. Neurosci. Methods 172(1), 137–141 (2008). JNMEDT 0165-0270 10.1016/j.jneumeth.2008.04.012 18501969 PMC3902859

[30] Hu S. et al., “Changes in cerebral morphometry and amplitude of low-frequency fluctuations of BOLD signals during healthy aging: correlation with inhibitory control,” Brain Struct. Funct. 219(3), 983–994 (2014). 23553547 10.1007/s00429-013-0548-0 PMC3760988

[r31] Zuo X. N. et al., “The oscillating brain: complex and reliable,” Neuroimage 49(2), 1432–1445 (2010). NEIMEF 1053-8119 10.1016/j.neuroimage.2009.09.037 19782143 PMC2856476

[r32] Tian X. et al., “Assessment of trait anxiety and prediction of changes in state anxiety using functional brain imaging: a test-retest study,” Neuroimage 133, 408–416 (2016). NEIMEF 1053-8119 10.1016/j.neuroimage.2016.03.024 27001499

[33] Han Y. et al., “Frequency-dependent changes in the amplitude of low-frequency fluctuations in amnestic mild cognitive impairment: a resting-state fMRI study,” Neuroimage 55(1), 287–295 (2011). NEIMEF 1053-8119 10.1016/j.neuroimage.2010.11.059 21118724

[34] Song X.-W. et al., “REST: a toolkit for resting-state functional magnetic resonance imaging data processing,” PLoS One 6(9), e25031 (2011). POLNCL 1932-6203 10.1371/journal.pone.0025031 21949842 PMC3176805

[35] Yan C.-G. Zang Y.-F., “DPARSF: a MATLAB toolbox for ‘pipeline’ data analysis of resting-state fMRI,” Front. Syst. Neurosci. 4, 13 (2010). 10.3389/fnsys.2010.00013 20577591 PMC2889691

[36] Jia X. Z. et al., “RESTplus: an improved toolkit for resting-state functional magnetic resonance imaging data processing,” Sci. Bull. 64(14), 953–954 (2019). 10.1016/j.scib.2019.05.008 36659803

[37] Zhang H. et al., “Functional connectivity as revealed by independent component analysis of resting-state fNIRS measurements,” Neuroimage 51(3), 1150–1161 (2010). NEIMEF 1053-8119 10.1016/j.neuroimage.2010.02.080 20211741

[38] Molloy E. K. Meyerand M. E. Birn R. M., “The influence of spatial resolution and smoothing on the detectability of resting-state and task fMRI,” Neuroimage 86, 221–230 (2014). NEIMEF 1053-8119 10.1016/j.neuroimage.2013.09.001 24021836 PMC5736131

[39] Mennes M. et al., “Inter-individual differences in resting-state functional connectivity predict task-induced BOLD activity,” Neuroimage 50(4), 1690–1701 (2010). NEIMEF 1053-8119 10.1016/j.neuroimage.2010.01.002 20079856 PMC2839004

[40] http://www.nitrc.org/projects/nirskit/.

[41] Cope M. Delpy D. T., “System for long-term measurement of cerebral blood and tissue oxygenation on newborn infants by near infrared transillumination,” Med. Biol. Eng. 26(3), 289–294 (1988). 10.1007/BF02447083 2855531

[42] Singh A. K. et al., “Spatial registration of multichannel multi-subject fNIRS data to MNI space without MRI,” Neuroimage 27(4), 842–851 (2005). NEIMEF 1053-8119 10.1016/j.neuroimage.2005.05.019 15979346

[43] Jasper H., “The ten twenty electrode system of the international federation,” Electroencephalogr. Clin. Neurophysiol. 10, 371–375 (1958). ECNEAZ 0013-4694 10590970

[44] Cui X. Bray S. Reiss A. L., “Functional near infrared spectroscopy (NIRS) signal improvement based on negative correlation between oxygenated and deoxygenated hemoglobin dynamics,” Neuroimage 49(4), 3039–3046 (2010). NEIMEF 1053-8119 10.1016/j.neuroimage.2009.11.050 19945536 PMC2818571

[45] Fishburn F. A. et al., “Temporal derivative distribution repair (TDDR): a motion correction method for fNIRS,” Neuroimage 184, 171–179 (2019). NEIMEF 1053-8119 10.1016/j.neuroimage.2018.09.025 30217544 PMC6230489

[46] Biswal B. et al., “Functional connectivity in the motor cortex of resting human brain using echo-planar MRI,” Magn. Reson. Med. 34(4), 537–541 (1995). MRMEEN 0740-3194 10.1002/mrm.1910340409 8524021

[47] Lu H. et al., “Synchronized delta oscillations correlate with the resting-state functional MRI signal,” Proc. Natl. Acad. Sci. U. S. A. 104(46), 18265–18269 (2007). 10.1073/pnas.0705791104 17991778 PMC2084331

[48] Yuen N. H. et al., “Intrinsic frequencies of the resting-state fMRI signal: the frequency dependence of functional connectivity and the effect of mode mixing,” Front. Neurosci. 13, 900 (2019).1662-453X 10.3389/fnins.2019.00900 31551676 PMC6738198

[49] Gagnon L. et al., “Improved recovery of the hemodynamic response in diffuse optical imaging using short optode separations and state-space modeling,” Neuroimage 56(3), 1362–1371 (2011). NEIMEF 1053-8119 10.1016/j.neuroimage.2011.03.001 21385616 PMC3085546

[r50] Zhang Q. Strangman G. E. Ganis G., “Adaptive filtering to reduce global interference in non-invasive NIRS measures of brain activation: how well and when does it work?” Neuroimage 45(3), 788–794 (2009). NEIMEF 1053-8119 10.1016/j.neuroimage.2008.12.048 19166945 PMC2671198

[r51] Sato T. et al., “Reduction of global interference of scalp-hemodynamics in functional near-infrared spectroscopy using short distance probes,” Neuroimage 141, 120–132 (2016). NEIMEF 1053-8119 10.1016/j.neuroimage.2016.06.054 27374729

[r52] Emberson L. L. et al., “Isolating the effects of surface vasculature in infant neuroimaging using short-distance optical channels: a combination of local and global effects,” Neurophotonics 3(3), 031406 (2016). 10.1117/1.NPh.3.3.031406 27158631 PMC4835587

[53] Yücel M. A. et al., “Short separation regression improves statistical significance and better localizes the hemodynamic response obtained by near-infrared spectroscopy for tasks with differing autonomic responses,” Neurophotonics 2(3), 035005 (2015). 10.1117/1.NPh.2.3.035005 26835480 PMC4717232

[54] Yamada T. Umeyama S. Matsuda K., “Separation of fNIRS signals into functional and systemic components based on differences in hemodynamic modalities,” PLoS One 7(11), e50271 (2012). POLNCL 1932-6203 10.1371/journal.pone.0050271 23185590 PMC3501470

[55] Van Den Heuvel M. P. Pol H. E. H., “Exploring the brain network: a review on resting-state fMRI functional connectivity,” Psiquiatr. Biol. 18(1), 28–41 (2011). 10.1016/j.psiq.2011.05.001 20471808

[56] Cordes D. et al., “Mapping functionally related regions of brain with functional connectivity MR imaging,” Am. J. Neuroradiol. 21(9), 1636–1644 (2000). 11039342 PMC8174861

[57] Molavi B. et al., “Analyzing the resting state functional connectivity in the human language system using near infrared spectroscopy,” Front. Hum. Neurosci. 7, 921 (2014). 10.3389/fnhum.2013.00921 24523685 PMC3905209

[58] Nguyen T. et al., “Investigation of brain functional connectivity in patients with mild cognitive impairment: a functional near-infrared spectroscopy (fNIRS) study,” J. Biophotonics 12(9), e201800298 (2019). 10.1002/jbio.201800298 30963713

[59] Latora V. Marchiori M., “Economic small-world behavior in weighted networks,” Eur. Phys. J. B 32, 249–263 (2003). EPJBFY 1434-6028 10.1140/epjb/e2003-00095-5

[60] Latora V. Marchiori M., “Efficient behavior of small-world networks,” Phys. Rev. Lett. 87(19), 198701 (2001). PRLTAO 0031-9007 10.1103/PhysRevLett.87.198701 11690461

[61] Newman M. E. J., “Finding community structure in networks using the eigenvectors of matrices,” Phys. Rev. E 74, 036104 (2006). 10.1103/PhysRevE.74.036104 17025705

[62] Wang J. et al., “GRETNA: a graph theoretical network analysis toolbox for imaging connectomics,” Front. Hum. Neurosci. 9, 386 (2015). 10.3389/fnhum.2015.00386 26175682 PMC4485071

[r63] van den Heuvel M. P. et al., “Proportional thresholding in resting-state fMRI functional connectivity networks and consequences for patient-control connectome studies: issues and recommendations,” Neuroimage 152, 437–449 (2017). NEIMEF 1053-8119 10.1016/j.neuroimage.2017.02.005 28167349

[64] Cai L. Dong Q. Niu H., “The development of functional network organization in early childhood and early adolescence: a resting-state fNIRS study,” Dev. Cognit. Neurosci. 30, 223–235 (2018). 10.1016/j.dcn.2018.03.003 29631206 PMC6969083

[65] van Wijk B. C. M. Stam C. J. Daffertshofer A., “Comparing brain networks of different size and connectivity density using graph theory,” PLoS One 5(10), e13701 (2010). POLNCL 1932-6203 10.1371/journal.pone.0013701 21060892 PMC2965659

[66] Genvese C. R. Lazar N. A. Nichols T., “Thresholding of statistical maps in functional neuroimaging using the false discovery rate,” Neuroimage 15(4), 870–878 (2002). NEIMEF 1053-8119 10.1006/nimg.2001.1037 11906227

[67] Benjamini Y. Hochberg Y., “Controlling the false discovery rate: a practical and powerful approach to multiple testing,” J. R. Stat. Soc. Ser. B 57(1), 289–300 (1995). JSTBAJ 0035-9246 10.1111/j.2517-6161.1995.tb02031.x

[68] R Foundation, “R project for statistical computing,” www.r-project.org.

[69] Okamoto M. et al., “Three-dimensional probabilistic anatomical cranio-cerebral correlation via the international 10–20 system oriented for transcranial functional brain mapping,” Neuroimage 21(1), 99–111 (2004). NEIMEF 1053-8119 10.1016/j.neuroimage.2003.08.026 14741647

[70] Okamoto M. Dan I., “Automated cortical projection of head-surface locations for transcranial functional brain mapping,” Neuroimage 26(1), 18–28 (2005). NEIMEF 1053-8119 10.1016/j.neuroimage.2005.01.018 15862201

[71] Neuroimaging tools & resource collaboratory (NITRC), “MRIcroGL,” https://www.nitrc.org/projects/mricrogl.

[72] NITRC, “Surf Ice,” https://www.nitrc.org/projects/surfice/.

[73] Tian F. Lin Z.-J. Liu H., “EasyTopo: a toolbox for rapid diffuse optical topography based on a standard template of brain atlas,” Proc. SPIE 8578, 85782J (2013). PSISDG 0277-786X 10.1117/12.2003907

[74] Xia M. Wang J. He Y., “BrainNet viewer: a network visualization tool for human brain connectomics,” PLoS One 8(7), e68910 (2013). POLNCL 1932-6203 10.1371/journal.pone.0068910 23861951 PMC3701683

[75] Friston K. J. et al., “Statistical parametric maps in functional imaging: a general linear approach,” Hum. Brain Mapp. 2(4), 189–210 (1994). HBRME7 1065-9471 10.1002/hbm.460020402

[76] Pinti P. et al., “Current status and issues regarding pre-processing of fNIRS neuroimaging data: an investigation of diverse signal filtering methods within a general linear model framework,” Front. Hum. Neurosci. 12, 1–21 (2019). 10.3389/fnhum.2018.00505 PMC6336925 30687038

[77] Pfeifer M. D. Scholkmann F. Labruyère R., “Signal processing in functional near-infrared spectroscopy (fNIRS): methodological differences lead to different statistical results,” Front. Hum. Neurosci. 11, 1–12 (2018). 10.3389/fnhum.2017.00641 PMC5766679 29358912

[78] Klein F. Kranczioch C., “Signal processing in fNIRS: a case for the removal of systemic activity for single trial data,” Front. Hum. Neurosci. 13, 1–23 (2019). 10.3389/fnhum.2019.00331 31607880 PMC6769087

[79] Friston K. J., “Functional and effective connectivity: a review,” Brain Connect. 1(1), 13–36 (2011). 10.1089/brain.2011.0008 22432952

[80] Vergotte G. et al., “Dynamics of the human brain network revealed by time-frequency effective connectivity in fNIRS,” Biomed. Opt. Express 8(11), 5326 (2017). BOEICL 2156-7085 10.1364/BOE.8.005326 29188123 PMC5695973

[r81] Liu Z. et al., “Effective connectivity analysis of the brain network in drivers during actual driving using near-infrared spectroscopy,” Front. Behav. Neurosci. 11, 211 (2017). 10.3389/fnbeh.2017.00211 29163083 PMC5671603

[82] Hu Z. Lam K. F. Yuan Z., “Effective connectivity of the fronto-parietal network during the tangram task in a natural environment,” Neuroscience 422, 202–211 (2019). 10.1016/j.neuroscience.2019.09.021 31682954

[83] Kohno S. et al., “Removal of the skin blood flow artifact in functional near-infrared spectroscopic imaging data through independent component analysis,” J. Biomed. Opt. 12(6), 062111 (2007). JBOPFO 1083-3668 10.1117/1.2814249 18163814

[84] von Lühmann A. et al., “A new blind source separation framework for signal analysis and artifact rejection in functional near-infrared spectroscopy,” Neuroimage 200, 72–88 (2019). NEIMEF 1053-8119 10.1016/j.neuroimage.2019.06.021 31203024

[85] Zhang Y. et al., “Eigenvector-based spatial filtering for reduction of physiological interference in diffuse optical imaging,” J. Biomed. Opt. 10(1), 011014 (2005). JBOPFO 1083-3668 10.1117/1.1852552 15847580

[86] Belouchrani A. Abed-Meraim K. Cardoso J.-F., “Second-order blind separation of temporally correlated sources,” in Proc. Int. Conf. Digital Sig. Proc., pp. 346–351 (1993).

[87] Moreau E., “A generalization of joint-diagonalization criteria for source separation,” IEEE Trans. Signal Process. 49(3), 530–541 (2001). ITPRED 1053-587X 10.1109/78.905873

[88] Hyvarinen A., “Fast and robust fixed-point algorithms for independent component analysis,” IEEE Trans. Neural Networks 10(3), 626–634 (1999). ITNNEP 1045-9227 10.1109/72.761722 18252563

[89] Bell A. J. Sejnowski T. J., “An information-maximization approach to blind separation and blind deconvolution,” Neural Comput. 7(6), 1129–1159 (1995). NEUCEB 0899-7667 10.1162/neco.1995.7.6.1129 7584893

[90] Li X.-L. Adali T., “Independent component analysis by entropy bound minimization,” IEEE Trans. Signal Process. 58(10), 5151–5164 (2010). ITPRED 1053-587X 10.1109/TSP.2010.2055859

